# Tissue‐Agnostic Cellular Morphometric Biomarkers for Risk‐Adapted Management Across Gastrointestinal Precancerous Lesions and Cancers

**DOI:** 10.1002/advs.77353

**Published:** 2026-08-25

**Authors:** Pin Wang, Chengfei Jiang, April W. Mao, Qi Sun, Yijun Lu, Jingjing Wei, Jamie L. Inman, Susan E. Celniker, Antoine M. Snijders, David W. Threadgill, Allan Balmain, Bo Hang, Hang Chang, Lei Wang, Jian‐Hua Mao

**Affiliations:** ^1^ Department of Hepatobiliary Oncology Zhongshan Hospital Fudan University Shanghai China; ^2^ Liver Cancer Institute Zhongshan Hospital Key Laboratory of Carcinogenesis and Cancer Invasion (Ministry of Education) Fudan University Shanghai China; ^3^ National Clinical Research Center for Interventional Medicine Shanghai China; ^4^ Department of Gastroenterology Nanjing Drum Tower Hospital Affiliated Hospital of Medical School Nanjing University Nanjing Jiangsu China; ^5^ Biological Systems and Engineering Division Lawrence Berkeley National Laboratory Berkeley California USA; ^6^ Department of Mathematics University of California Los Angeles Los Angeles California USA; ^7^ Department of Pathology Nanjing Drum Tower Hospital The Affiliated Hospital of Nanjing University Medical School Nanjing Jiangsu China; ^8^ Department of Hepatobiliary Surgery and Liver Transplantation Liver Cancer Institute Zhongshan Hospital Fudan University Key Laboratory of Carcinogenesis and Cancer Invasion Ministry of Education Shanghai China; ^9^ Department of Gastroenterology Nanjing Drum Tower Hospital Clinical College of Nanjing University of Chinese Medicine Nanjing Jiangsu China; ^10^ Berkeley Biomedical Data Science Center Lawrence Berkeley National Laboratory Berkeley California USA; ^11^ Department of Nutrition Texas A&M University College Station Texas USA; ^12^ Department of Molecular and Cellular Medicine and Department of Biochemistry & Biophysics Texas A&M University College Station Texas USA; ^13^ Helen Diller Family Comprehensive Cancer Center University of California San Francisco San Francisco USA; ^14^ Department of Biochemistry and Biophysics University of California San Francisco San Francisco California USA

**Keywords:** artificial intelligence, computational biology, gastroenterology, genetics, oncology, pathology, precision medicine, risk assessment, risk stratification, tumor microenvironment

## Abstract

While precision oncology increasingly adopts tissue‐agnostic paradigms, current strategies remain heavily reliant on molecular alterations, with limited relevance to early‐stage cancers and precancerous lesion management. Here we present an unsupervised and interpretable artificial intelligence framework that defines tissue‐agnostic cellular morphometric biomarkers (CMBs) capturing conserved tumor microenvironment (TME) architectures associated with cancer progression across gastrointestinal (GI) organs. Discovered from colorectal cancer whole‐slide images and validated in gastric and esophageal malignancies within a multi‐center cohort of 2,602 patients, a 13‐CMB signature demonstrates robust cross‐GI tissue transferability and prognostic impact. Importantly, beyond prognostic value in pan‐GI cancers, the 13‐CMB signature enables risk stratification of precancerous lesions and early‐stage cancers, addressing a key unmet need in clinical decision‐making where molecular profiling is often impractical or insufficient. The CMB‐based risk scores support risk‐adapted management, including individualized surveillance intervals and tailored intervention strategies. Integrative analyses using bulk and single‐cell RNA sequencing and immunohistochemistry profiling show that CMBs correspond to biologically interpretable morphological states of the TME, including immune‐excluded and stromal‐dominant architectures. Together, this study establishes a biologically transparent, tissue‐agnostic CMB framework that links conserved TME organization to clinically actionable risk assessment, providing a scalable approach for early cancer management and precision oncology.

## Graphical abstract

An interpretable, unsupervised artificial intelligence framework identifies a 13‐cellular morphometric biomarker (CMB) signature from routine H&E whole‐slide images. Validated across 2,602 patients, the fixed signature generalizes across colorectal, gastric, and esophageal tissues without retraining, stratifies prognosis and precancerous lesion risk, and links morphological risk states to immune exclusion and stromal fibrosis, providing an interpretable platform for scalable risk stratification and precision oncology.

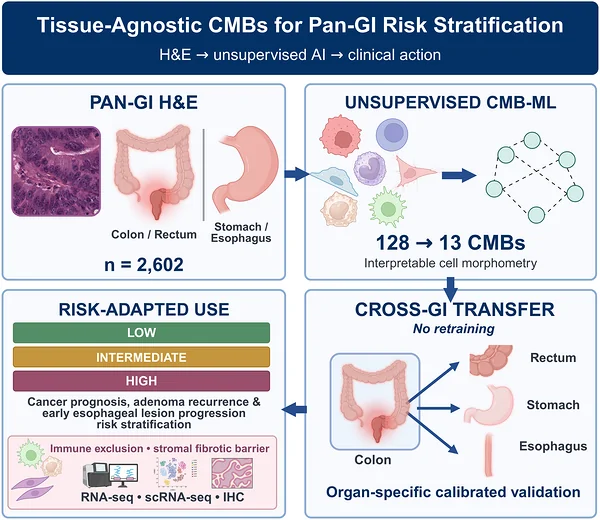

**Abbreviations**
AI: artificial intelligence
CAP: Colon Adenomatous Polyps
CMB: cellular morphometrics biomarker
CMBRG: CMB risk group
CMBRS: CMB risk score
CRC: Colorectal Cancer
DT: Drum Tower Hospital
EAC: Esophageal adenocarcinoma
EEL: Early Esophageal Lesion
ESCA: esophageal carcinoma
ESCC: Esophageal squamous cell carcinoma
GI: gastrointestinal
H&E: hematoxylin and eosin
HGIN: High‐Grade Intraepithelial Neoplasia
IHC: Immunohistochemistry
LGIN: Low‐Grade Intraepithelial Neoplasia
OS: Overall survival
scRNA‐seq: Single cell RNA sequencing
TCGA‐COAD: The Cancer Genome Atlas – Colon Adenocarcinoma
TCGA‐ESCA: The Cancer Genome Atlas – Esophageal Carcinoma
TCGA‐READ: The Cancer Genome Atlas – Rectum Adenocarcinoma
TCGA‐STAD: The Cancer Genome Atlas – Stomach Adenocarcinoma
WSIs: whole‐slide images

## 1 Introduction

The past decades of cancer research and clinical oncology have witnessed a fundamental paradigm shift toward characterizing tumors at cellular and molecular levels, paving the way for tissue‐agnostic therapies. Emerging evidence validates the efficacy of targeted agents across diverse tumor types. (e.g., nivolumab, a PD‐1 inhibitor [1]; trastuzumab deruxtecan, a HER2‐targeted antibody‐drug conjugate [2, 3]; and olaparib, a poly (ADP‐ribose) polymerase inhibitor effective in patients with germline *BRCA1/2* mutations [4]). These breakthroughs leverage shared molecular profiles regardless of the organ of origin, challenging the traditional organ‐centric classification of cancer. Concurrently, major cancer types, including breast [5], lung [6], brain [7], and gastric cancers [8, 9], are being increasingly subdivided into distinct molecular subgroups to optimize treatment strategies. Recognizing this transformative trend, regulatory agencies like the U.S. Food and Drug Administration (FDA) are actively establishing guidelines for tissue‐agnostic drug development [10, 11], signaling a potential restructuring of oncology based on molecular landscapes rather than anatomical location.

However, tumor heterogeneity extends far beyond molecular alterations and molecular subtypes identified in these extensive characterization efforts. It is intrinsically encoded in tissue histology, where the spatial arrangement of the cytoplasm, nucleus, organelles, and extracellular matrix reflects the dynamic and complex interplay of the tumor microenvironment (TME) [12, 13]. With the adoption of digital workflows, artificial intelligence (AI) has revealed the possibility of identifying such biomarkers from whole‐slide images (WSIs) [14]. Previous efforts have extensively documented how specific histological features correlate with biological behaviors. For instance, quantitative analyses of nuclear atypia and chromatin patterns have provided critical insights into tumor aggressiveness [15, 16, 17]; assessments of stromal ratio and fibrosis have highlighted the barrier role of the extracellular matrix [18, 19, 20]; while characterization of immune infiltration patterns has been established as a robust prognosticator reflecting anti‐tumor immunity [21, 22, 23, 24]. While these morphological phenotypes often represent evolutionarily conserved biological states shared across organs, they remain largely underexploited in the current molecular‐centric precision oncology framework. Furthermore, the widespread implementation of comprehensive molecular profiling faces practical barriers, including high costs, limited accessibility, and long turnaround times. These limitations not only impede its global application in primary care but also exacerbate health disparities [25]. In contrast, hematoxylin and eosin (H&E)‐stained histopathological slides remain the gold standard for cancer diagnosis, which underscores the immense potential of histology‐based biomarkers as a cost‐effective and time‐saving approach for precision oncology.

This gap is particularly consequential in gastrointestinal (GI) oncology, where effective risk assessment of precancerous lesions and early‐stage cancers is central to improving outcomes. Despite a global rise in colorectal cancer (CRC) incidence [26], including a concerning increase among adults younger than 50 years [27], progress in risk‐adapted management strategies has lagged [25]. As a result, the limited ability to accurately stratify progression risk in early disease frequently leads to overtreatment of indolent lesions through unnecessary invasive interventions, while aggressive lesions may remain under‐recognized, resulting in missed opportunities for timely and potentially curative treatment.

In this study, we applied our previously established Cellular Morphometric Biomarker via Machine Learning (CMB‐ML) framework from the whole H&E staining slides. Building on prior demonstrations of CMBs’ clinical value in organ‐specific contexts, we here extend this interpretable AI approach to derive GI tissue‐agnostic cellular morphometric biomarkers (CMBs) across pan‐ GI malignancies (Figure 1). Unlike supervised models constrained by predefined labels, our framework autonomously uncovers conserved morphological patterns from a discovery cohort (TCGA‐COAD) and validates their transferability in a large, multi‐center pan‐GI cohort encompassing precancers and early‐stage cancers. By combining these evolutionarily conserved CMBs with clinical variables, we develop a robust CMB risk score (CMBRS) and risk group (CMBRG) stratification system using tissue‐agnostic CMBs with organ‐specific calibration, enabling quantitative risk assessment in both early‐stage lesions and advanced diseases. This framework not only enhances prognostic accuracy but also informs risk‐adapted management strategies, supporting individualized surveillance and intervention for precancerous and early‐stage GI cancers. Also, we show that these CMBs act as interpretable “morphological imprints” of underlying tumor biology, capturing tumor microenvironment (TME) states such as immune exclusion and stromal barrier intensity, confirmed through comprehensive multi‐omics integration, including bulk RNA sequencing, single‐cell RNA sequencing, and multiplex immunohistochemistry. Together, our study establishes CMBs as a scalable, biologically transparent, GI tissue‐agnostic alternative to molecular testing, bridging computational histopathology with clinically actionable decision‐making in precision oncology.

**FIGURE 1:**
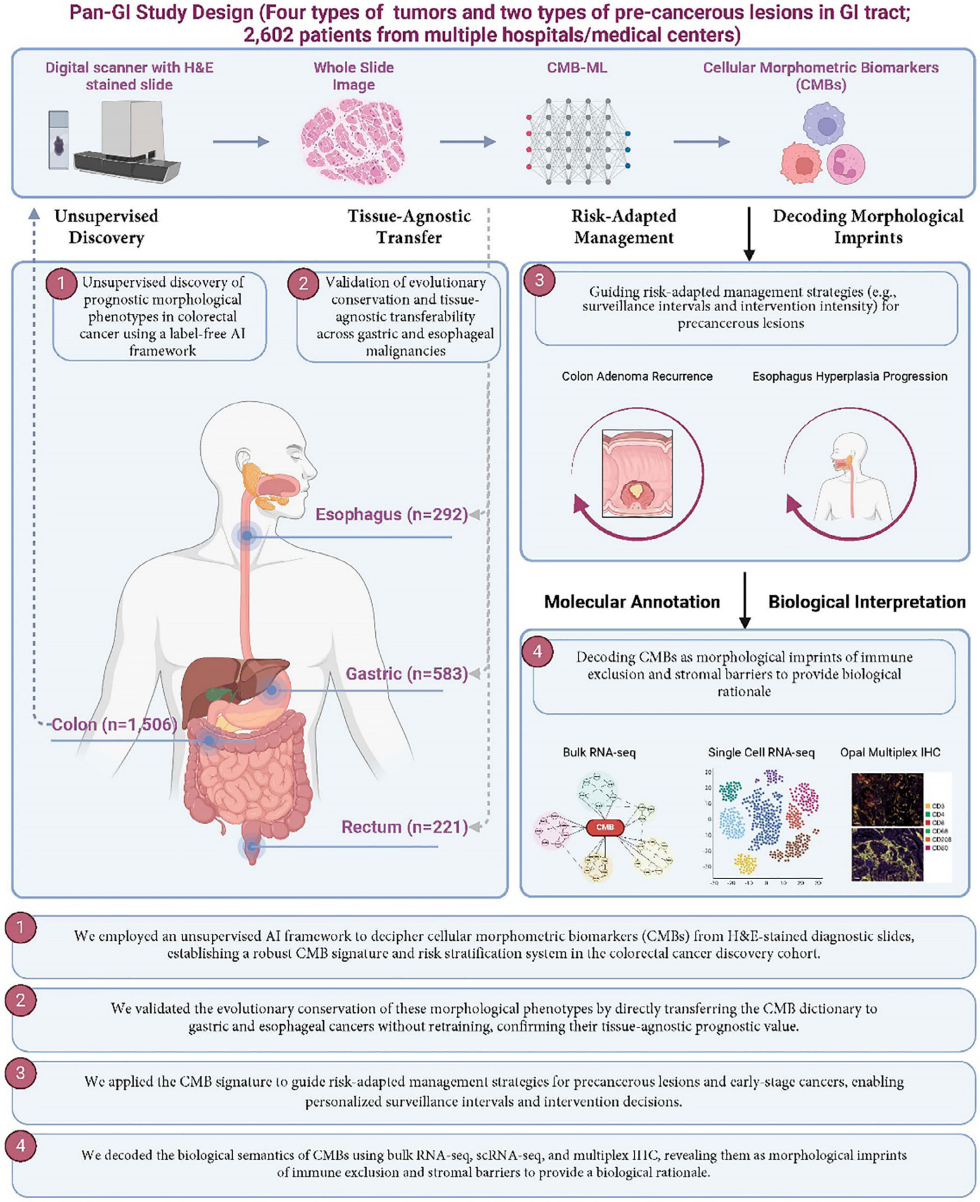
Pan‐GI study design and the purpose of each step. A four‐stage study workflow for unsupervised CMB discovery, tissue‐agnostic transferability validation, risk‐adapted management in precancerous lesions, and biological decoding of morphological imprints.

## 2 Materials and Methods

### 2.1 Human Cohorts

The human gastrointestinal cancer and precancerous cohorts included in this study consist of 4 cohorts with 1,118 patients collected from 38 clinical sites in the Cancer Genome Atlas (TCGA) project (Tables S1–S4), and 5 cohorts with 1,484 patients collected from the Nanjing Drum Tower Hospital (DT), the Affiliated Hospital of Nanjing University Medical School (Figures S1–S5 and Tables S5–S11). Detailed study design, inclusion, and exclusion criteria for all cohorts are provided in Methods S1.

### 2.2 Unsupervised Discovery of Interpretable CMBs and Risk Stratification System

To decipher the intrinsic morphological heterogeneity of the TME in an unbiased manner, we employed the CMB‐ML framework approach [15, 16, 28]. Unlike supervised deep learning methods that rely on human annotations, this framework utilizes stacked predictive sparse decomposition (PSD) [29] for unsupervised dictionary learning. Specifically, we defined 128 CMBs from cellular objects extracted from WSIs of H&E‐stained tissue histological sections in the TCGA‐COAD cohort. In the CMB‐ML pipeline, we used a single network layer with 128 CMBs and a sparsity constraint of 30 at a fixed random sampling rate of 1,000 cellular objects per WSI from the cohort. Crucially, to ensure biological interpretability, each individual CMB was refined during training as the combinatorial quantification and sparse activation of fifteen critical morphometric properties, including nuclear size, chromatin density, smoothness of nuclear contour, and the background properties around nuclear regions, detail morphometric information and definition of which were presented in the previous study [16]; altogether, these CMBs were optimized to reconstruct the tissue heterogeneity. After training, the pretrained CMB‐ML model reconstructed each cellular region as a sparse combination of 128 predefined CMBs, and each patient was represented as an aggregation of all delineated cellular objects belonging to the same patient. The experimental settings were identical to those used in our previous study [29] to maintain a reconstruction error of less than 10% during training.

To translate the high‐dimensional morphological features into a clinically actionable tool, we performed a multistep feature selection process. The prognostic effect of high or low levels of each CMB on overall survival (OS) was assessed by Kaplan‐Meier analysis (survminer package in R, version 0.4.8) and log‐rank test (survival package in R, version 3.2‐3), where the TCGA‐COAD cohort was divided into two groups (i.e., CMB‐high and CMB‐low groups) based on each CMB (cut‐off estimated using survminer package in R, version 0.4.8). The set of CMBs as a prognostic signature was selected using a stepwise multivariate Cox proportional hazards (CoxPH) regression model, including CMBs with a significant effect on poor outcome events. The construction of the CMB Risk Score (CMBRS) is defined below, where the coefficients of the final CMBs as categorical variables were obtained from the multivariate Cox PH regression analysis:

$$\begin{array}{ccc} \mathrm{CMBRS}\; & = & \sum_{i=1}^{N}\left(\mathrm{coefficient}\;\mathrm{of}\;\mathrm{CMB}\_\mathrm{Categor}y^{i}\right)\\ & & *\left(\mathrm{CMB}\_\mathrm{Categor}y^{i}\right) \end{array}$$

where N is the number of final CMBs that were independently and significantly associated with poor outcome events, and $\mathrm{CMB}\_\mathrm{Categor}y^{i}$ is the category of the i^th^ CMB (i.e., CMB‐high = 1; CMB‐low = 0). After CMBRS construction, the retrospective cohort was divided into a high‐risk group (top 1/3), intermediate‐risk group (middle 1/3), and low‐risk group (bottom 1/3) based on the CMBRS cut‐offs between high/intermediate and intermediate/low, which were recorded and fixed for the CMBRG model. During the hospital validation study, the cut‐off points for both CMB and CMBRS remained unchanged.

### 2.3 GI Tissue‐Agnostic Feature Transfer and Organ‐Specific Calibration of Risk Model

To evaluate the GI tissue‐agnostic nature of the identified morphological phenotypes, the CMB dictionary (comprising 128 basis functions) learned exclusively from the TCGA‐COAD cohort was transferred directly to the gastric (STAD) and esophageal (ESCA) cancer cohorts. Crucially, the feature extraction process remained fixed, meaning the AI model was not retrained on the new tissue types, demonstrating that the morphological patterns discovered in colon cancer are evolutionarily conserved across GI malignancies. However, acknowledging the inherent biological heterogeneity in baseline prognosis across different organs, we performed organ‐specific calibration for risk stratification. Specifically, while the CMB features and quantifications were fixed, the optimal cut‐off points for individual CMBs and the CMBRS were calibrated within each tumor type's respective discovery cohort (e.g., TCGA‐STAD for gastric cancer) using the same maximization of rank statistics strategy. Once established, these organ‐specific cut‐offs and scoring models were strictly fixed and applied to independent external validation cohorts (e.g., Nanjing Drum Tower cohorts) to ensure rigorous evaluation of clinical utility.

### 2.4 Multivariate Cox Proportional Hazards Regression Modeling

To evaluate the independent prognostic significance of the CMBRS in both discovery and validation cohorts, multivariate Cox proportional hazards models were constructed including CMBRS and clinical factors (*e.g*., age, sex, tumor stage, etc.). The CMBRS was included as a continuous or dichotomized variable, and adjusted hazard ratios (HRs) were reported with corresponding 95% confidence intervals. The proportional hazards assumption was assessed using Schoenfeld residuals.

### 2.5 Multi‐Omics Decoding of CMB Biological Functions

For each CMB, Pearson correlation analysis was performed among all TCGA samples described above to determine the genes significantly associated with each CMB (*p* < 0.05). Enrichment analysis was conducted using the online databases GO and KEGG to annotate the biological function of the genes related to different CMB (statistical significance: adjusted p‐value < 0.05, cell component enrichment: Q value < 0.05, biological process enrichment Q value <0.2, molecular function enrichment: Q value <0.2, KEGG enrichment Q value < 0.2). The results of these enrichment analyses were used to draw a network diagram of the same function shared with different CMB. Functional networks were then created in Cytoscape (version 3.10.1) based on the results of enrichment analysis for Cellular Components, Molecular Function, Biological Process, and KEGG.

### 2.6 scRNA Sequencing and Data Processing

In this study, we prospectively collected 17 colon cancer samples to validate our findings using scRNA sequencing, where all patients had been diagnosed with colon cancer through biopsy before resection (Table S12). Details are provided in Methods S2 and S3.

### 2.7 Multiplexed Immunohistochemical (IHC) Staining

Details are provided in Method S4.

### 2.8 Nomogram With Calibration Curve

To evaluate the capability of CMBRG in predicting prognosis, nomogram models for each of the nine cohorts containing all the independent variables related to prognosis or recurrence were constructed using the rms package (v6.7.1). All models were validated by a calibration curve through 3‐fold cross‐validation.

### 2.9 Statistical Analysis

Statistical analysis was performed using the R software (version 4.2.2). The Kaplan‐Meier plot with the log‐rank test was used to evaluate the prognostic value of CMBRG. A Multivariable CoxPH regression model was employed to demonstrate the significant and independent prognostic value of the CMBRS by adjusting for other important clinical factors. Pearson or Spearman correlation analysis was used to evaluate the association between the CMBRS and other clinical or pathological factors. The receiver operating characteristic curve (ROC) model was employed to describe the performance of the prognostic model with or without CMBRS (statistical significance: *p* < 0.05).

### 2.10 Patient and Public Involvement

Patients were not involved in the development of the research question and outcome measures.

## 3 Results

### 3.1 Unsupervised Discovery of Prognostic CMBs in Colorectal Cancer

To decipher the intrinsic morphological heterogeneity of gastrointestinal tumors in an unbiased manner, we designed a comprehensive and international multicenter study with multi‐step sequential evaluation and validation (Figure 1). In the discovery phase, we employed the unsupervised CMB‐ML pipeline on whole‐slide images (WSIs) from the TCGA‐COAD cohort [16]. This framework autonomously learned a dictionary of 128 Cellular Morphometric Biomarkers (CMBs) (Figure 2A). Each CMB was mathematically deconvoluted into a sparse combination of 15 interpretable morphometric properties, such as nuclear size, chromatin density, and contour smoothness, effectively capturing the intrinsic “morphological vocabulary” of the tumor microenvironment.

**FIGURE 2:**
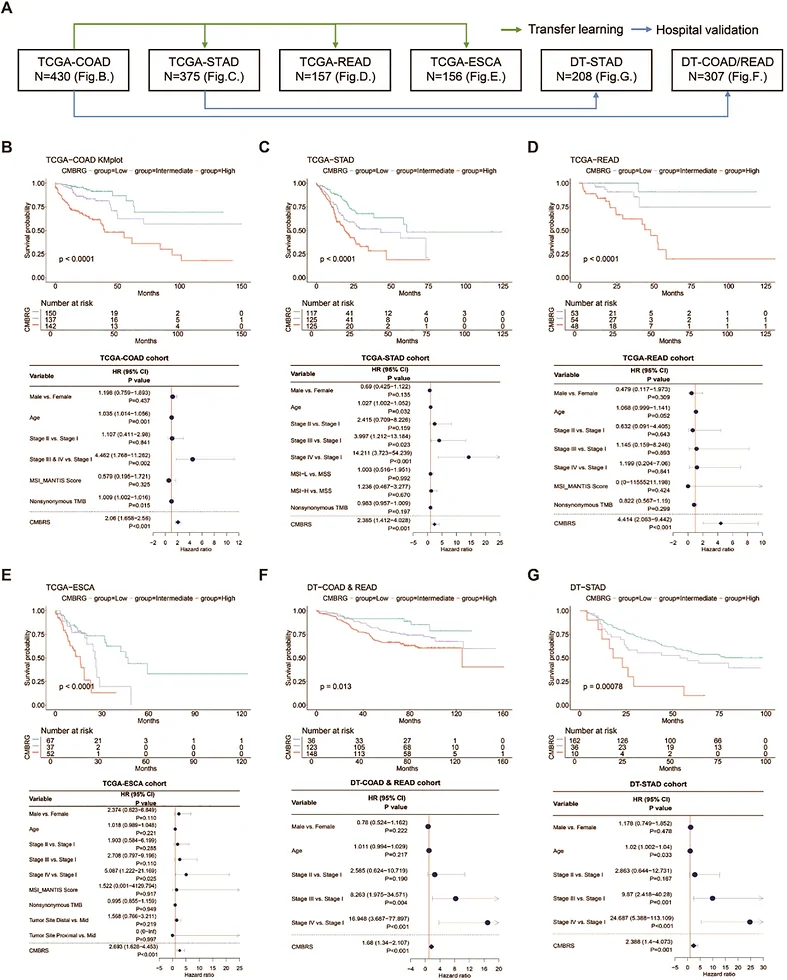
Discovery and tissue‐agnostic validation of the 13‐CMB signature across GI cancers. (A) Overview of study cohorts; (B‐G) Prognostic risk stratification (Top panel) and multivariate analysis using CMB Risk Score (CMBRS) (Bottom panel) on overall survival in (B) TCGA‐COAD cohort; (C) TCGA‐STAD cohort; (D) TCGA‐READ cohort; (E) TCGA‐ESCA cohort; (F) Drum Tower Hospital (DT)‐ COAD&READ cohort; and (G) DT‐STAD cohort. The *p* values in K‐M plots were obtained using log‐rank test, and the *p* values in forest plots were obtained using multivariate Cox Proportional‐Hazards regression analysis.

Through systematic survival screening, we found that these morphological patterns were clinically relevant, with 75 of the 128 CMBs showing significant prognostic impact (*p* < 0.05). To distill these patterns into a clinically actionable tool, we applied multivariate modeling to identify a robust 13‐CMB signature that captured the most critical morphological features driving tumor progression (Figure 2B and Figure S5). Based on this signature, we constructed the CMB Risk Score (CMBRS), a quantitative metric of morphological malignancy. CMB coefficients for cancer‐specific CMBRS calculation summarized in Table S13. We then stratified patients into distinct risk groups (high, top third; intermediate, middle third; low, bottom third) based on CMBRS cut‐offs. Kaplan‐Meier analysis revealed a striking stratification of outcomes, with the high‐risk group exhibiting significantly inferior overall survival compared to the low‐risk group (*p* < 0.0001, Figure 2B, top panel). Importantly, multivariate Cox regression demonstrated that the CMBRS remained a strong prognostic factor independent of standard clinical variables, including TNM stage (Hazard Ratio [HR] = 2.08, *p* = 0.001, Figure 2B, bottom panel). This suggests that CMBs are able to capture orthogonal biological information, likely reflecting intrinsic tumor aggressiveness or microenvironmental states, that is not accounted for by traditional staging methods. Furthermore, the multimodal integration of the CMBRS with clinical factors significantly enhanced prognostic power compared to clinical factors or CMBRS alone (*p* < 0.05; Figure S6A and S7A and Table S14), with calibration curves confirming the model's robust predictive reliability (Figure S7B).

### 3.2 Evolutionary Conservation and GI Tissue‐Agnostic Transferability of CMBs Across GI Malignancies

To test the hypothesis that the identified morphological phenotypes represent evolutionarily conserved biological states shared across GI malignancies, we directly transferred the CMB dictionary learned exclusively from colon cancer to rectal (TCGA‐READ), gastric (TCGA‐STAD), and esophageal (TCGA‐ESCA) cancer cohorts (Figure 2A). Crucially, to rigorously validate the GI tissue‐agnostic nature of these biomarkers, the feature extraction and quantification framework remained fixed without retraining. We only performed organ‐specific calibration of risk thresholds to account for the inherent heterogeneity in baseline prognosis across different GI tumor types. Strikingly, the 13‐CMB signature maintained powerful prognostic stratification in all three cancer types (*p* < 0.0001; HR = 4.414 in TCGA‐READ; HR = 2.385 in TCGA‐STAD; and HR = 2.693 in TCGA‐ESCA; Figure 2C–E). Notably, this prognostic value remained robust independent of histological subtypes (esophageal adenocarcinoma vs. squamous cell carcinoma) in esophageal carcinoma (Figure S8), suggesting that CMBs capture fundamental properties of tumor aggressiveness that transcend specific histological lineages.

Beyond independent prognostic value, we further evaluated whether CMBs could enhance current clinical staging systems. In all three cohorts (READ, STAD, ESCA), the integration of the CMBRS with standard clinical factors significantly improved prognostic accuracy compared to unimodal clinical models, as evidenced by increased AUC and C‐index values (*p* < 0.05; Figures S6B–D and S7C,E,G and Table S14). To facilitate clinical application, we constructed multimodal nomograms for each cancer type. The calibration curves demonstrated excellent agreement between predicted and observed survival probabilities (Figure S7D,F,H), confirming that the CMB signature can be seamlessly integrated into existing clinical workflows to provide robust, tissue‐agnostic risk assessment.

To further confirm the robustness and generalizability of these findings in a real‐world clinical setting, we validated the 13‐CMB signature in two independent external cohorts from Nanjing Drum Tower Hospital (DT), comprising 208 gastric and 307 colorectal cancer patients (Figures S1 and S2). We found that CMBRS was a significant prognostic factor independent of clinical factors in both cohorts (*p* = 0.001, *p* < 0.001; Figure 2F, G; Figure S6E,F and S7I–L). The generalizability and reproducibility of the CMBRS based on the same CMBs with organ‐specific calibration indicated that the 13‐CMB signature is a robust tissue‐agnostic biomarker for pan‐GI cancer.

### 3.3 CMB‐Guided Risk‐Adapted Management in Precancerous Lesions and Early‐Stage Cancers

To test whether the morphological programs driving lethality in advanced cancer constitute a fundamental path of malignancy, we applied the CRC‐derived CMB signature directly to precancerous lesions (colon adenomas) and early‐stage cancers (early esophageal lesions) without retraining. This GI tissue‐agnostic approach tests the hypothesis that high‐risk early lesions already harbor the morphological imprints, including immune exclusion and stromal rigidity, typically observed in aggressive diseases. By assessing the morphological resemblance of early lesions to lethal cancers, we aimed to stratify patients according to progression risk, enabling timely, risk‐adapted clinical intervention. Effective secondary prevention relies on precise risk stratification to tailor surveillance intervals and intervention intensity. To support clinical decision‐making in this context, we evaluated the utility of the 13‐CMB signature in two critical scenarios: colon adenomatous polyps (CAPs) and Early Esophageal Lesions (EELs) (Figure 3A and Figures S3 and S4). In the context of surveillance strategy for colon adenomas, Kaplan‐Meier analysis demonstrated that the CMBRG effectively discriminated patients with high recurrence risk from those with an indolent course (Figure 3B). Specifically, patients in the low‐risk group exhibited a significantly prolonged median recurrence‐free survival (42.5 months) compared to the high‐risk group (23.7 months). This distinction suggests that low‐risk patients may benefit from extended surveillance intervals, whereas high‐risk patients require more frequent monitoring. Crucially, multivariate analysis confirmed that the CMBRS remained an independent risk factor after adjusting for standard clinical variables, including age, sex, location, and adenoma count (Figure 3C). Furthermore, the integration of CMBRS with clinical factors significantly outperformed clinical factors alone in risk prediction (Figure 3D and Figure S6G), and the resulting nomogram provided an accurate and convenient tool for clinical deployment (Figure 3E). These findings were robustly validated in an independent DT‐CAP‐Validation cohort (Figures 3F–I and Figure S6H), confirming the reliability of CMBs in guiding post‐polypectomy surveillance.

**FIGURE 3:**
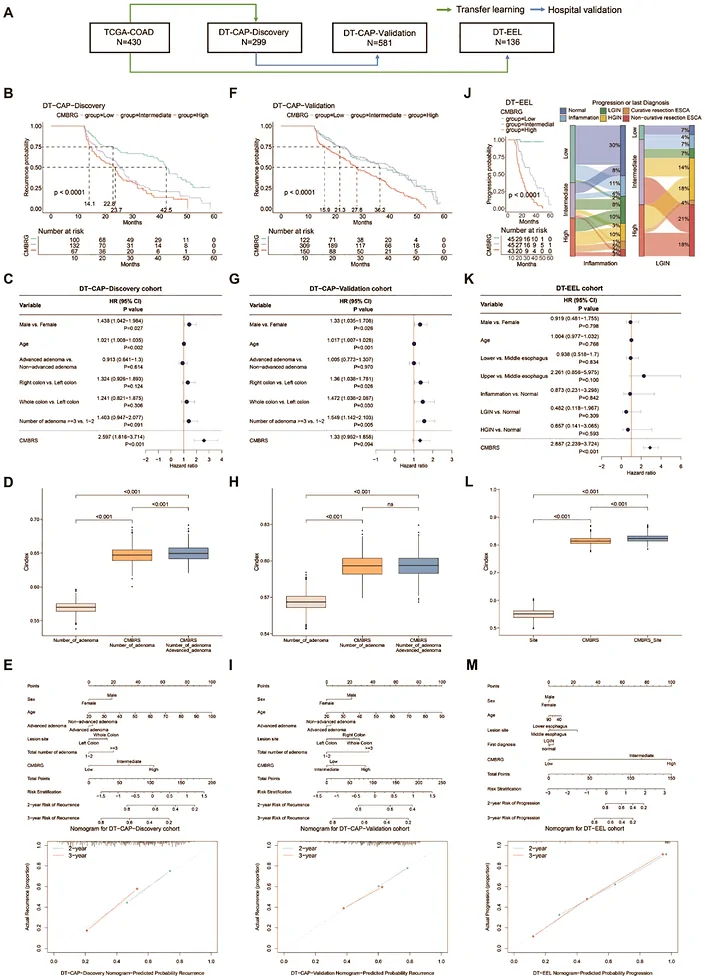
Clinical application of the 13‐CMB signature for risk assessment in precancerous lesions and early‐stage cancer. (A) Overview of the study cohorts, including DT‐CAP‐Discovery, DT‐CAP‐Validation, and DT‐EEL. (B‐E) In the DT‐CAP‐Discovery cohort, (B) CMBRG stratified patients into risk groups with significantly different recurrence‐free survival. (C) CMBRS demonstrated independent prognostic value after adjusting for clinical variables. (D) The combination of CMBRS with clinical variables significantly improved the prognostic power. (E) The nomogram constructed on CMBRG and clinical variables accurately predicted the 3‐year and 5‐year recurrence or progression probabilities. (F‐I) In the DT‐CAP‐Validation cohort, (F) CMBRG stratified patients into risk groups with significantly different recurrence‐free survival. (G) CMBRS demonstrated independent prognostic value after adjusting for clinical variables. (H) The combination of CMBRS with clinical variables significantly improved the prognostic power. (I) The nomogram constructed on CMBRG and clinical variables accurately predicted the 3‐year and 5‐year recurrence or progression probabilities. (J‐M) In the DT‐EEL cohort, (J, left panel) CMBRG stratified patients into risk groups with significantly different progression probabilities. (J, middle/right panels) The progression of patients with esophageal inflammation or LGIN across low, intermediate, and high CMBRG in the DT‐EEL cohort. (K) CMBRS demonstrated independent predictive value after adjusting for clinical variables. (L) The combination of CMBRS with clinical variables significantly improved the predictive power. (M) The nomogram constructed on CMBRG and clinical variables accurately predicted the 3‐year and 5‐year progression probabilities. The *p* values in K‐M plots were obtained using the log‐rank test, the *p* values in forest plots were obtained using multivariate Cox Proportional‐Hazards regression analysis, and the *p* values in boxplots were obtained using the Mann‐Whitney nonparametric test.

Similarly, in early esophageal lesions, where the decision between endoscopic resection and active surveillance is often challenging, the CMBRG successfully stratified patients into distinct progression risk groups independent of clinical factors (Figure 3J,K). The CMBRS demonstrated superior predictive capability over clinical factors alone, with further accuracy gains achieved through multimodal integration (Figure 3L,M and Figure S6I and Table S14). Notably, the CMBRG identified distinct biological trajectories: over 95% of patients with esophageal inflammation in the low‐risk group never progressed, with nearly 70% showing regression to normal tissue (Figure 3J, middle panel). This finding supports a conservative monitoring strategy for this subgroup to avoid overtreatment. In sharp contrast, 100% (28/28) of inflammation patients in the high‐risk group experienced disease progression, suggesting an urgent need for early intervention. This dichotomy was even more pronounced in patients with Low‐Grade Intraepithelial Neoplasia (LGIN), where none of the low‐risk patients showed progression, whereas 100% (11/11) of the high‐risk patients progressed (Figure 3J, right panel).

Taken together, these findings demonstrate that the clinical utility of the 13‐CMB signature is not just for prognosis, but as a decision‐support tool for risk‐adapted management, enabling personalized surveillance and intervention strategies for patients with precancerous and early‐stage lesions.

### 3.4 CMBs as Morphological Imprints of Heterogeneous Tumor and Its Microenvironments

To provide a biological rationale for the tissue‐agnostic predictive power of CMBs, we next deciphered their underlying biological functions. First, we identified genes that were transcriptionally correlated to each CMB in the four TCGA datasets (Table S15). We then constructed enrichment networks of Gene Ontology (GO) and KEGG pathways (Figure 4A and Figure S9). These analyses revealed that 11 of the 13 signature CMBs were significantly enriched for five biological functions (Figure 4A), confirming they serve as morphological surrogates for cellular proliferation, immune microenvironment, and DNA repair. Specifically, CMB12, CMB67, CMB76, CMB86, CMB97, and CMB120 are associated with the formation of cell junctions, cytoskeleton, and extracellular matrix, suggesting a fibrotic barrier phenotype. CMB9 and CMB85 are associated with cell replication and DNA repair. CMB29 is associated with both innate and adaptive immunities, and CMB47 is associated with the regulation of adaptive immunity.

**FIGURE 4:**
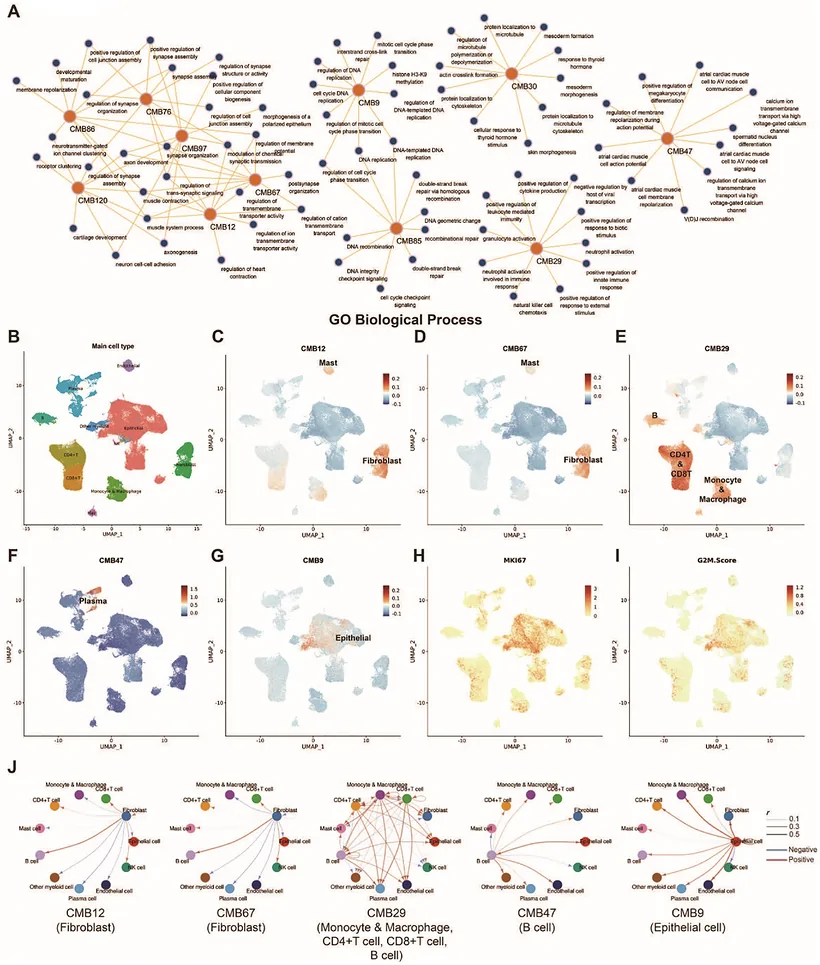
Biological interpretation of CMBs at bulk RNA‐seq and scRNA‐seq levels. (A) Biological process in GO terms significantly enriched by the correlated genes of 11 out of 13 CMBs. (B‐I) UMAP visualization of single‐cell sequencing results, highlighting cell clusters and prognostically representative CMBs marker expression. (J) Cell‐cell communication networks of major cell types associated with each representative CMB. Each network illustrates the significant interactions between the primary cell type of a given CMB (labeled below each network) and other major immune and stromal cell types. Nodes represent different cell types, and edges indicate significant ligand‐receptor interactions. Arrows denote the direction of signaling, from ligand‐expressing source cells to receptor‐expressing target cells. The color of the edges represents the type of correlation, with red indicating positive associations and blue indicating negative associations. The edge width is proportional to the correlation strength, as defined by the Spearman correlation coefficient (r).

To further resolve the cellular heterogeneity underlying these phenotypes, we conducted CMB profiling in combination with scRNA‐seq of colon cancers prospectively collected from 17 patients in a double‐blind manner. Among these patients, none were diagnosed with hereditary nonpolyposis colorectal cancer or had a family history of colon cancer, and only one patient was identified as microsatellite unstable (i.e., MLH1 and PMS2 deficiencies) using IHC analysis. After the quality control procedure, we clustered 106,515 cells into 11 cell groups and found a significant difference in the composition of the different cell types (*p* < 0.001; Figure 4B and Figure S10). Next, we estimated the genes transcriptionally correlated with each CMB in these cell types (Figure 4C–G and Figure S11) to decipher their cellular identity. The genes associated with CMB9, CMB85, and CMB104 were predominantly expressed in tumor cells (Figure 4G and Figure S11B,F), where CMB9 captured tumor proliferation (Figure 4H,I); while the genes associated with CMB12, CMB67, CMB76, CMB86, CMB87, CMB97, and CMB120 were predominantly expressed in fibroblasts (Figure 4C, D and Figure S11A,C–E,G). Notably, the genes associated with CMB29, CMB86, CMB87, CMB97, and CMB120 were predominantly expressed in T lymphocytes (Figure 4E and Figure S11C—E,G). Among these, CMB29 and CMB87 were also strongly associated with monocytes and macrophages (Figure 4E and Figure S11D), and CMB29, CMB97, and CMB120 were expressed in B cells (Figure 4E and Figure S11E,G). Additionally, the genes associated with CMB47 were predominantly expressed in plasma cells (Figure 4F), and a broad cluster of stromal CMBs (CMB12, 67, 86, 87, 97, 120) showed predominant expression in mast cells (Figure 4C,D and Figure S11C–E,G).

Moreover, we used CellChat analysis to reveal distinct cell‐type interactions in each CMB (Figure 4J and Figure S12). For example, CMB9 demonstrated epithelial cell‐centered interactions, highlighting its role in epithelial‐stromal crosstalk. CMB29 displayed the highest interaction density, involving monocytes/macrophages, CD4+ T cells, CD8+ T cells, and fibroblasts, suggesting extensive immune‐stromal crosstalk (“immune‐hot”). In contrast, CMB30 exhibited a fibroblast‐centered network with moderate immune cell involvement. Additionally, CMB86 showed strong interactions between fibroblasts, mast cells, and T cells, further emphasizing the complex interplay between stromal and immune components. These findings indicate that CMBs capture distinct functional heterogeneity within the tumor microenvironment, serving as specific morphological indicators of immune exclusion versus immune activation.

Furthermore, we verified the association between CMBs and immune cell types by scRNA‐seq (Figure 4B–G and Figure S11) and multiplexed IHC staining on FFPE samples from the DT‐COAD‐scRNASeq cohort (Figure 5A,B), and demonstrated consistent associations for most of the CMBs (Figure 5B, marked by circles). Notably, the biological interpretation of CMBs by scRNA‐seq and multiplex IHC staining is highly consistent with the pathological interpretation by pathologists (Figure 6), including the CMBs that captured distinct cell types such as tumor cells (e.g., CMB12, CMB85, CMB104), fibroblasts (e.g., CMB67, CMB76, CMB86, CMB87, CMB9), immune cells (e.g., CMB29 and CMB47), and immune‐ and stroma‐rich microenvironments (e.g., CMB97, CMB76, CMB120). In addition, CMBs in ESCC (Figure S13) capture highly consistent pathological concepts compared to those in adenocarcinomas (Figure 6), which explains the tissue‐agnostic power of CMBs across histological subtypes. Taking all the findings from bulk RNA‐seq, scRNA‐seq, Opal staining, and pathological interpretation, we conclude that CMBs represent consistent biological and functional interpretability.

**FIGURE 5:**
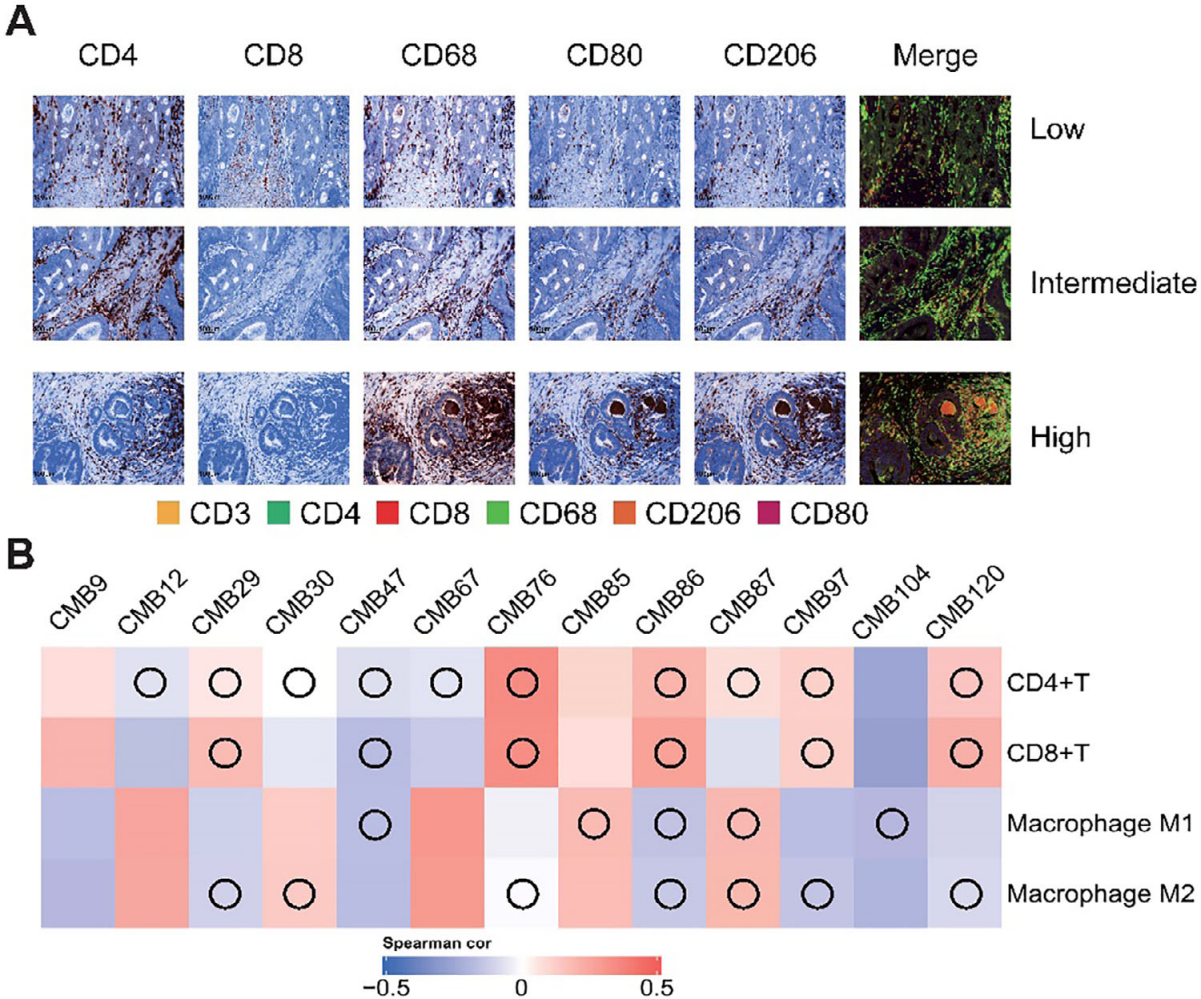
Immune Cell Characterization and Correlation with CMBs. (A) Multiplex immunofluorescence illustrates the five known markers of immune cells, CD8+T(CD3+/CD8+), CD4+T(CD3+/CD4+), M1 macrophage (CD68+/CD80+), and M2 macrophage (CD68+/CD206+). (B) The heatmap illustrates the correlation between CMBs and immune cells quantified from the multiplex immunofluorescence. A box with a circle indicates the consistent interpretation of CMB between scRNA‐seq and OPAL staining.

**FIGURE 6:**
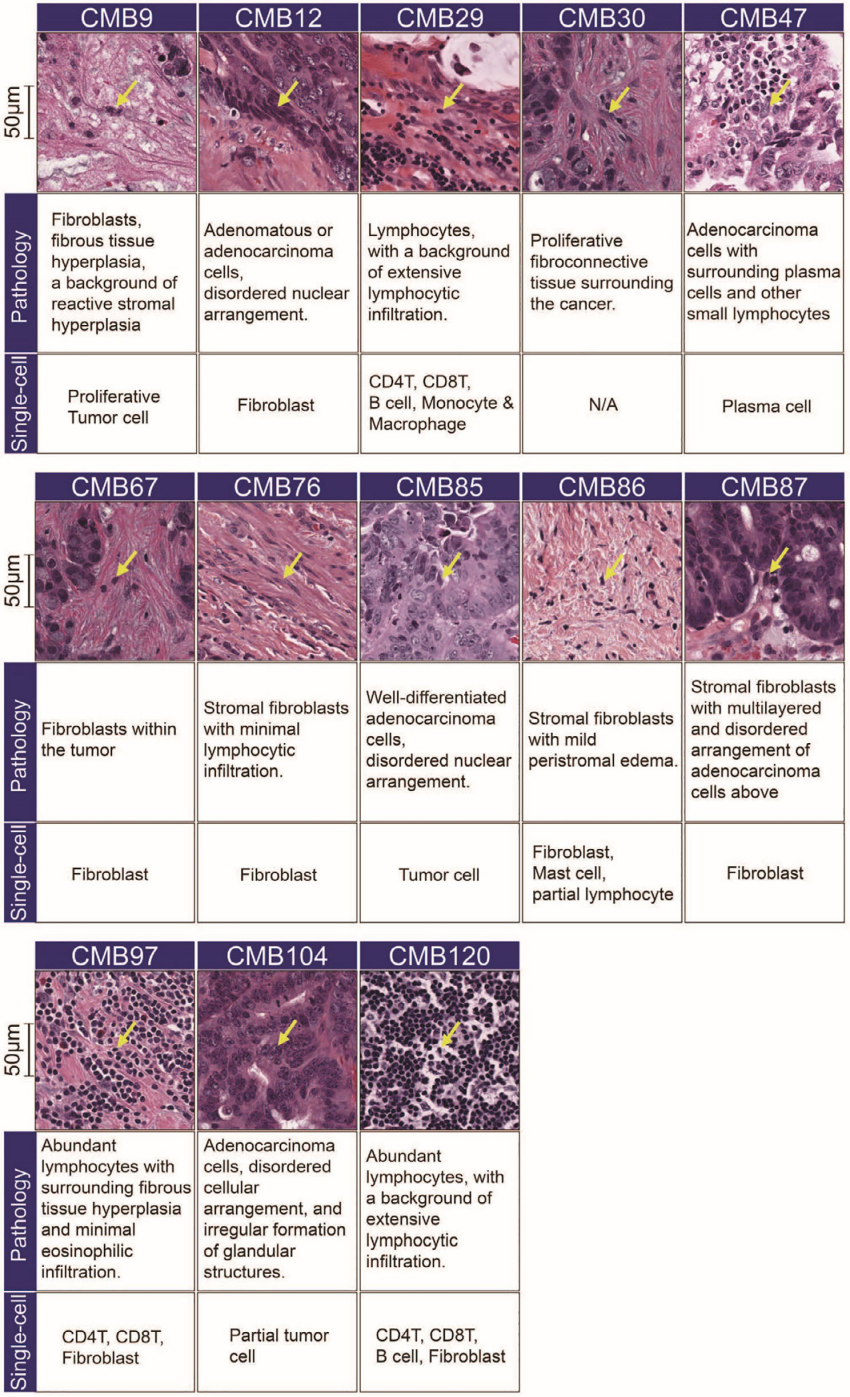
Representative examples (highlighted with yellow arrows) and local pathological neighborhood of CMBs, demonstrating the consistent interpretability of CMBs across pathologic and scRNA‐seq levels.

## 4 Discussion

Tissue‐agnostic precision oncology represents a fundamental paradigm shift, aiming to restructure cancer classification based on shared biological characteristics rather than anatomical origin. While molecular biomarkers have successfully driven this transformation in targeted therapies, the vast potential of histology‐based tissue‐agnostic markers remains largely untapped. In this study, we identified and deciphered a novel set of CMBs that characterize the intrinsic morphological heterogeneity of the TME directly from WSIs, and further demonstrated the prognostic values of CMBRS and CMBRG based on tissue‐agnostic CMBs with organ‐specific calibration. Unlike supervised approaches constrained by human annotations, our unsupervised discovery framework allowed us to identify evolutionarily conserved morphological phenotypes shared across GI malignancies [15, 16, 17]. This study represents a systematic effort to discover, validate, and biologically interpret these GI tissue‐agnostic CMBs at scale, integrating computational pathology with tumor biology to guide risk‐adapted clinical oncology.

Our results indicate that CMBs are not merely statistical prognosticators but serve as interpretable “morphological imprints” of specific biological states. Through comprehensive multi‐omics validation, we confirmed that these morphological patterns spatially map to distinct microenvironmental phenotypes, particularly immune exclusion and stromal barrier intensity. Specifically, we found that high‐risk CMBs were densely populated by myofibroblasts and marked by extensive extracellular matrix remodeling, a pattern associated with reduced T‐cell infiltration into tumor nests and consistent with an immune‐excluded phenotype. This finding aligns with the “immune‐excluded” phenotype described in recent immuno‐oncology studies, which is increasingly recognized as a primary driver of resistance to immune checkpoint inhibitors. By morphologically quantifying this specific barrier state, CMBs effectively capture the spatial architecture of immune evasion mechanisms that are often missed by bulk genomic profiling. This biological transparency distinguishes our approach from “black box” deep learning, providing a mechanistic rationale for the universal predictive power of CMBs across different GI tumor types.

A persistent challenge in oncology lies in the secondary prevention and management of precancerous lesions, where current guidelines often result in overtreatment and significant resource strain [30]. For instance, in current clinical practice, patients with colorectal adenomas are uniformly suggested to undergo intensive colonoscopies post‐polypectomy, yet clinical data indicate that only ∼6% of them eventually develop CRC [30, 31, 32]. This discrepancy poses a major challenge: how to identify the minority at high risk without subjecting the majority to unnecessary, invasive, and costly procedures. Addressing this, our study demonstrates the utility of CMBs for risk‐adapted management. The CMBRS effectively stratified recurrence risk in colorectal adenoma, identifying a low‐risk subgroup with significantly prolonged recurrence‐free survival (approx. 40 vs. 25 months). This stratification supports the refinement of surveillance schedules, enabling extended intervals for low‐risk patients to optimize healthcare resources, while ensuring intensive monitoring for high‐risk individuals. Crucially, as a histology‐based tool, the CMB system is cost‐effective and globally scalable, offering a practical alternative to genomic profiling, particularly in primary care settings where sequencing is often inaccessible.

Furthermore, our framework offers a transformative solution for the personalized management of Early Esophageal Lesions (EELs). Currently, the management of these lesions is often standardized, lacking precision tools to predict individual outcomes. Unlike traditional diagnostics that assess only the current disease state [33, 34, 35], our model predicts the biological trajectory of the lesion. The CMBRS revealed striking dichotomies in progression risk: over 95% of low‐risk patients with esophageal inflammation never progressed, and remarkably, over 60% naturally recovered to normal tissue. This finding strongly supports a conservative monitoring strategy for low‐risk patients, sparing them from overtreatment and procedure‐related morbidity. In sharp contrast, 100% of high‐risk patients progressed, indicating the necessity for early intervention, such as endoscopic submucosal dissection (ESD), even before invasive carcinoma manifests. This capability to distinguish aggressive from indolent lesions at diagnosis directly addresses the clinical requirement for precision intervention, demonstrating that in the early‐stage setting, risk stratification itself is the decisive factor for clinical management, independent of systemic therapy.

We acknowledge certain limitations in our study. First, while our retrospective multi‐center validation ensures robustness, future studies should pursue prospective evaluations to integrate this signature into clinical workflows. Second, our current study focused on prognosis and risk stratification for early management. The findings support the potential utility of CMBs for risk stratification and warrant further prospective validation before informing clinical decision‐making. While this addresses the priorities of secondary prevention, future investigations will explore the predictive value of CMBs for therapeutic response in advanced settings. Given that CMBs effectively capture immune‐excluded phenotypes, they hold significant promise as companion diagnostics to guide immunotherapy or stromal‐targeting treatments in future clinical trials. Finally, while scRNA‐seq and mIHC analyses successfully map specific cell types to CMBs (e.g., CMB9 with proliferating tumor cells, CMB29 with immune infiltrates), we acknowledge that correlation does not imply causation. It remains to be definitively proven whether the observed morphological features are directly driven by these cell types, or if they are secondary epiphenomena resulting from broader microenvironmental signaling.

In summary, this study integrates unsupervised morphological discovery with rigorous multi‐omics validation to establish a tissue‐agnostic biomarker system. By uncovering novel CMBs that capture fundamental histological features and defined biological functions across multiple GI cancers and precancerous lesions, we provide a biological rationale for digital pathology in precision oncology. Our findings highlight that these evolutionarily conserved morphological imprints provide a robust basis for risk stratification, facilitating a shift toward risk‐adapted precision oncology that spans from early detection to advanced disease management.

## Author Contributions

P.W., J‐H.M. and L.W. Conceptualized and Designed the study. P.W. and A.W.M. Led the Model Construction and Formal Analysis from Public cohorts. P.W. and C.F.J. Led the Independent Hospital Validation, processed the Raw Data and Performed Formal Analysis in Hospital Validation cohorts. Q.S. Provided Pathological interpretation. P.W., C.F.J., A.W.M., Q.S., Y.J.L, J.J.W., J.L.I., S.E.C., A.M.S., D.W.T., A.B., B.H., H.C., L.W. and J‐H.M. were involved in the critical review of the data and/or interpretation of results. P.W., B.H., J‐H.M. and H.C. drafted the manuscript. All authors edited, reviewed, revised, and approved the manuscript text.

## Funding

This work was supported by the National Natural Science Foundation of China (Grant No. 82272952), the Natural Science Foundation of Jiangsu Province for Excellent Young Scholars (Grant No. BK20220094), and China Postdoctoral Science Foundation (Grant No. 2022M721579).

## Ethics Statement

The collection and research use of human tissue specimens and associated clinical data were approved by the Institutional Review Board of Nanjing Drum Tower Hospital, the Affiliated Hospital of Nanjing University Medical School (Approval No. 2024‐900). Written informed consent was obtained from all participants prior to tissue collection. This study adhered to the principles of the Declaration of Helsinki (2013) and followed the institutional research code.

## Conflicts of Interest

The authors declare no conflicts of interest.

## Supporting information




**Supporting File 1**: advs77353‐sup‐0001‐SuppMat.xlsx.

**Supporting File 2**: advs77353‐sup‐0002‐SuppMat.docx.

**Supporting File 3**: advs77353‐sup‐0003‐SupData.xlsx.

## Data Availability

Whole slide Images and Clinical Data For the TCGA Cohort Were Downloaded from the TCGA GDC Portal (https://portal.gdc.cancer.gov/). Processed data, Including Clinical Information, CMB profiles, CMBRS, and CMBRG for both the TCGA and Drum Tower cohorts, Are Provided in the Data S1–S10. Raw digital Pathological Files from the Nanjing Drum Tower Hospital Are Not Currently Permitted in Public Repositories Due to Ongoing Institutional Discussions Regarding Ethical and Legal Implications.
