# Supplementary material for: Tissue‐Agnostic Cellular Morphometric Biomarkers for Risk‐Adapted Management Across Gastrointestinal Precancerous Lesions and Cancers
Source: Adv Sci (Weinh). 2026 Aug 25;13(63):e77353. doi: 10.1002/advs.77353 (PMC13504578; doi:10.1002/advs.77353)
Supplement: Supplementary file 1 [file ADVS-13-e77353-s002.docx]

**Supplementary Methods**

**Supplementary Method S1. Human cohorts**

The TCGA-COAD cohort (Supplementary Table S1) included 430 patients diagnosed with colon adenocarcinoma, the TCGA-STAD cohort (Supplementary Table S2) included 375 patients diagnosed with stomach adenocarcinoma, the TCGA-READ cohort (Supplementary Table S3) included 157 patients diagnosed with rectal adenocarcinoma, and the TCGA-ESCA cohort (Supplementary Table S4) included 156 patients diagnosed with esophageal squamous cell carcinoma (ESCC, n=90) and esophageal adenocarcinoma (EAC, n=66). The inclusion criteria in TCGA cohorts are primary tumors with diagnostic slides and follow-up information.

The DT-COAD/READ cohort (Supplementary Figure S1, Supplementary Table S5 and S6) included 243 patients diagnosed with primary colon adenocarcinoma and 64 patients diagnosed with primary rectal adenocarcinoma, who received radical surgery between Jan. 2007~Dec.2015 at Nanjing Drum Tower Hospital. There were 177 (57.7%) male and130 (42.3%) female patients, with a median age of 60.0 years (range: 15-87 years). The complete cohort consisted of 1,135 patients with diagnostic slides and follow-up information, with 827 excluded due to loss of follow-up (n=542), lack of clinical information (n=152), diagnosis of other tumor between the follow-up duration (n=16), history of colorectal cancer before this study (n=21), no pathological slides or with insufficient quality slides (n=97).

The DT-STAD cohort (Supplementary Figure S2, Supplementary Table S7) included 208 patients diagnosed with primary gastric cancer, who received radical gastrectomy between Jan. 2009~Jun.2015 at Nanjing Drum Tower Hospital. There were 157 (75.5%) male and 51 (24.5%) female patients, with a median age of 62 years (range: 24-90 years). The complete cohort consisted of 1,198 patients with diagnostic slides and follow-up information, with 990 excluded due to loss of follow-up (n=766), lack of clinical information (n=67), history of gastric cancer before this study (n=77), no pathological slides or with insufficient quality slides (n=80).

The DT-CAP cohort (Supplementary Figure S3, Supplementary Tables S8-S10) included 880 patients who received colonoscopy between Jan. 2013~Dec. 2021 at Nanjing Drum Tower Hospital. There were 565 (64.2%) male and 315 (35.8%) female patients, with a median age of 60 years (range: 21-91 years). The complete cohort consisted of 8831 patients with diagnostic slides and follow-up information, with 7951 excluded due to non-adenoma disease (n=3,896), lost follow-up (n=3,692), lack of clinical information (n=135), history of colectomy (n=30), with hyperplastic polyps (n=116), no pathological slides or with insufficient quality slides (n=82). The cohort was divided into two sets, DT-CAP-Discovery set (n=299) whose patients were diagnosed from Jan. 2013 to Dec. 2016 and DT-CAP-Validation set (n=581) whose patients were diagnosed from Jan. 2017 to Dec. 2021. The recurrence was defined as the pathological confirmation of newly developed adenomatous polyps during patient follow-up.

The DT-EEL cohort (Supplementary Figure S4, Supplementary Table S11) included 136 patients with early neoplastic lesions diagnosed by gastroscopy between Jan. 2013~Dec. 2021 at Nanjing Drum Tower Hospital, where the H&E-stained slides at initial diagnose and the follow-up information are available. The initial diagnoses for these patients fall into four categories: normal (n=3), inflammation (n=96), low-grade intraepithelial neoplasia (LGIN) (n=28), and high-grade intraepithelial neoplasia (HGIN) (n=9). There were 86 (66.2%) male and 44 (33.8%) female patients, with a median age of 63 years (range: 34-85 years). The complete cohort consisted of 704 patients, with 568 excluded due to lost follow-up (n=237), the interval between two inspections less than one year (n=297), diagnosis of other tumors within the follow-up duration (n=8), history of esophagectomy (n=3), no pathological slides or with insufficient quality slides (n=23). The progression was defined as histopathological advancement to a higher-grade lesion during patient follow-up.

The DT-COAD-scRNASeq cohort (Supplementary Table S12) included 20 patients with colon cancer and received radical surgery from Aug. 2023 to Oct. 2023. There were 10 (50.0%) male and 10 (50.0%) female patients, with a median age of 63.5 years (range:32-91 years). The complete cohort consisted of 17 patients with diagnostic slides, with 3 excluded due to insufficient sample quality for single cell sequencing (n=3).

The model development using the publicly available TCGA-COAD cohort was performed at Lawrence Berkeley National Laboratory. The hospital validation study was approved by the Institutional Review Board (IRB) (NO. 2024-900-01) and independently carried out at Nanjing Drum Tower Hospital.

**Supplementary Method S2. Preparation of single-cell suspensions and scRNA sequencing**

In this study we prospectively collected 17 colon cancer samples to validate our findings using scRNA sequencing, where all the patients have been diagnosed with colon cancer through biopsy before the resection. For isolation of colon cells, fresh intestines were collected from colorectal resection. Three biological replicates were included in each group. Intestines were dissected and washed to remove fecal content with pre-cold PBS for several times, minced into small pieces, and incubated with 0.5 mg/mL digestive enzymes for 30 min at 37℃ in a water bath. After centrifugation, supernatant was collected, and the pellet was further digested with 0.5 mg/ml digestive enzyme for another 30 min. After digestion for total 60 min, all the supernatant and precipitation were combined and filtered through a 70 μM cell strainer (BD Falcon, 352350). After centrifugation at 500 g for 5 min, the precipitation was further digested with 1 mL trypsin for 15 min. The supernatants were collected, then all the supernatant was filtered through 35 μM cell strainer (BD Falcon, 352235). After centrifugation at 500 g for 5 min, the cell precipitated was resuspension with PBS+0.01%BSA.

Single cell suspensions were loaded on 10x Genomics Chromium™ according to manufacturer’s protocol based on the 10x GEM Code proprietary technology. scRNA-seq libraries were prepared using 10x Genomics Chromium Next GEM Single Cell 3ʹ Kits v3.1 according to manufacturer’s protocol. Briefly, the initial step involves performing an emulsion where individual cells were isolated into droplets together with gel beads coated with unique primers bearing 10x Genomics cell barcodes, unique molecular identifiers (UMI), and poly(dT) sequences. Reverse transcription reactions were engaged to generate barcoded full-length cDNA followed by the disruption of emulsions using the recovery agent and cDNA clean up. Bulk cDNA was amplified and cleaned up. Sequencing libraries were constructed using the reagents from the 10x Genomics Chromium Next GEM Single Cell 3ʹ Kits v3.1, following these steps: (1) fragmentation, end repair, and a-tailing; (2) size selection with SPRI select; (3) adaptor ligation; (4) post ligation cleanup with SPRI select; (5) sample index PCR and cleanup with SPRI select beads. Indexed libraries were pooled according to number of cells and sequenced on a NovaSeq 6000 (Illumina) using paired-end 150 bp.

**Supplementary Method S3. scRNA-seq data processing**

The reads mapping and quality control steps were performed through Cellranger (v.7.1.0). The exclusion criteria of cells as below: the number of UMI > 500, percentage of mitochondria gene < 30% and percentage of hemoglobin gene < 5%. After exclusion, we used scDblFinder to detect and remove the cells identified as doublet. The total number of cells included into the final analysis was 106515. A seurat object was generated from the filtered scRNA-seq data using the package Seurat (v.4.1.3). Then we conducted the Single Cell Transform (SCT transform), Principal Component Analysis (PCA), graph-based clustering and Uniform Manifold Approximation and Projection (UMAP) to reduce the dimension information of the scRNA-seq data and clustered the cells into 27 subclusters and annotated the clusters with 11 main cell types with their feature genes. We applied chi-square test to evaluate the difference of cell composition among three CMBRGs and spearman correlation to identify the CMB associated with specific cell clusters. CellChat (v.1.6.1) was employed to describe the ligand-receptor interaction between each cell subclusters. For each CMB, Pearson correlation analysis was performed among all the TCGA pan-GI cohorts to determine the genes significantly associated with each CMB (Statistical significance: *P* value < 0.05). Using the *AddModuleScore* function from package Seurat, we added the score of each CMB based on the average expression level of CMB-related genes to every single cell. UMAP plot was deployed to illustrate the distribution of each CMB score in different cell clusters. The scores of proliferation related genes and G2/M phase related genes were estimated and illustrated using the similar approach.

**Supplementary Method S4. Multiplexed immunohistochemical (IHC) staining**

5μm Paraffin-embedded sections were deparaffinized with xylene and gradient ethanol solutions, and antigen retrieval was conducted via microwave treatment. Endogenous peroxidase was neutralized with endogenous peroxidase blocking solution (SP KIT-A1 Fuzhou Maixin Biotech. Co., Ltd.) that also blocked the binding of irrelevant antibodies. The primary antibodies series included anti-CD4 (Cat No. ab133616, dilution 1:500, Abcam), anti-CD8α (Cat No. 85336, dilution 1:200, Cell Signaling), anti-CD3 (Cat No. ab16669, dilution 1:150, Abcam), anti-CD68 (Cat No. 76437, dilution 1:400, Cell Signaling), anti-CD206 (Cat No. 91992, dilution 1:400, Cell Signaling) and anti- CD80 (Cat No. ab134120, dilution 1: 1000, Abcam) antibodies. Following the application of the Opal polymer HRP anti-rabbit secondary antibodies (Panovue), to detect the antibody staining, the tissues were incubated with one of the following fluorophores according to the manufacturer’s instructions: PPD 520, PPD 620, PPD 570, PPD 540, PPD 650 and PPD690 (dilution 1:100). The tissue section was then mounted in SlowFade Gold Antifade Reagent with DAPI (Thermo Fisher Scientific, Inc.). Whole slide tissue scanning was performed at 10× magnification using the Vectra Polaris System (Akoya Biosciences), and tumor area according to the H&E staining of the adjacent slide was performed at 20× magnification for subsequent analysis in Inform 2.6.0. The intensity of each fluorescein was deconvoluted from 8 randomly selected fields at 20× magnification. After the acquisition and deconvolution of fluorescence signal, we used spearman correlation to find the CMB related to specific cell cluster.

**Supplementary Figures**

**Supplementary Figure S1**

**Supplementary Figure S1.** **Illustrated flowchart for the sample collection of DT-COAD/READ cohort.**

**Supplementary Figure S2**

**Supplementary Figure S2. Illustrated flowchart for the sample collection of DT-STAD cohort.**

**Supplementary Figure S3**

**Supplementary Figure S3. Illustrated flowchart for the sample collection of DT-CAP cohort, where the discovery set consists of patients diagnosed before 2017, and the validation set consists of patients diagnosed in and after 2017.**

**Supplementary Figure S4**

**Supplementary Figure S4. Illustrated flowchart for the sample collection of the DT-EEL cohort.**

**Supplementary Figure S5**

**Supplementary Figure S5. Representative examples of each CMB and its local pathological environment.**

**Supplementary Figure S6**

**Supplementary Figure S6. Comparison of AUC Between CMBRG and Clinical Factors for Prognosis Prediction.** Time-dependent ROC curves comparing the AUC of the CMBRG model with other clinical factors, including staging and gene signature models, across various cohorts: (**A**) TCGA-COAD, (**B**) TCGA-STAD, (**C**) TCGA-READ, (**D**) TCGA-ESCA, (**E**) DT-STAD, (**F**) DT-COAD & READ, (**G**) DT-CAP-Discovery, (**H**) DT-CAP-Validation, and (**I**) DT-EEL.

**Supplementary Figure S7**

**Supplementary Figure S7. Comparison of prognostic power between clinical factors, CMBRS and the multimodal integration through C-Index, and the construction of multimodal nomograms for clinical deployment, where the C-Index was evaluated through bootstrapping strategy with 1000 iterations, and the nomograms were justified by calibration curves.** (**A, B**) TCGA-COAD, (**C, D**) TCGA-STAD, (**E, F**) TCGA-READ, (**G, H**) TCGA-ESCA, (**I, J**) DT-STAD, and (**K, L**) DT-COAD & READ. The CMBRG-based nomograms improve the predictive accuracy for 3-year and 5-year outcomes, as shown in calibration plots.

**Supplementary Figure S8**

**Supplementary Figure S8. Evaluation of the prognostic power of CMBRS/CMBRG across histology subtypes in the TCGA-ESCA cohort, where the TCGA-EAC and TCGA-ESCC cohorts consist of patients with adenocarcinoma and squamous cell carcinoma, respectively.** (**A-C**) CMBRS is an independent prognostic factor after adjusting for clinical factors in the (**A**) TCGA-ESCA; (**B**) TCGA-EAC; and (**C**) TCGA-ESCC cohorts. (**D-F**) In the TCGA-EAC cohort, (**D**) CMBRG stratified patients into risk groups with significantly different overall survival; (**E-F**) the integration of CMBRG with clinical factors significantly improved the prognostic power. (**G-I**) In the TCGA-ESCC cohort, (**G**) CMBRG stratified patients into risk groups with significantly different overall survival; (**H-I**) the integration of CMBRG with clinical factors significantly improved the prognostic power. P values in forest plots (**A-C**) were calculated using multivariate Cox Proportional-Hazards regression; P values in K-M plots (**D, G**) were calculated using log-rank test; P values in boxplots (**F, I**) were calculated using Mann-Whitney nonparametric test.

**Supplementary Figure S9**

**Supplementary Figure S9. Functional enrichment analysis of CMB-associated genes.** (**A**) KEGG pathway enrichment analysis for genes correlated with each CMB, showing pathways significantly enriched (*P* < 0.05). (**B**) GO term enrichment analysis of the cellular component category for genes correlated with each CMB (*P* < 0.05, *Q* < 0.2). (**C**) Top 10 molecular function GO terms enriched by genes correlated with each CMB, with significance thresholds of *P* < 0.05 and *Q* < 0.05. Genes were filtered using Spearman's correlation, and all enrichment analyses met the significance criteria (*P* < 0.05).

**Supplementary Figure S10**

**Supplementary Figure S10. Heatmap illustrating the expression of known marker genes in eleven clusters.**

**Supplementary Figure S11**

**Supplementary Figure S11. The single cell UMAP plots for additional CMBs that are not included in Figure 4.**

**Supplementary Figure S12**

**Supplementary Figure S12. Cell–cell communication networks for additional CMBs that are not included in Figure 4.**

**Supplementary Figure S13**

**Supplementary Figure S13. Representative examples of each CMB and its local pathological environment in ESCC from the TCGA-ESCA cohort.** NOTE: Consistency refers to similarity in CMBs between adenocarcinoma and ESCC; EMT refers to [epithelial-mesenchymal transition.](https://pmc.ncbi.nlm.nih.gov/articles/PMC7182759/)

**Supplementary Figure S14**

**Supplementary Figure S14. The calibration curves demonstrating the predictive accuracy of the nomogram for overall survival (OS) at 3-year and 5-year intervals across different cohorts**. (**A**) TCGA-COAD, (**B**) TCGA-STAD, (**C**) DT-STAD, and (**D**) DT-COAD & READ. The proximity of the curves to the diagonal line indicates the strong agreement between predicted probabilities and observed outcomes.

**Supplementary Tables**

**Supplementary Table S1. Baseline table of TCGA-COAD cohort.**

|  | **Low** | **Intermediate** | **High** | **Total** | ***P* value** |
| --- | --- | --- | --- | --- | --- |
|  | **N=150** | **N=137** | **N=143** | **N=430** |  |
| Sex [No. %] |  |  |  |  | 0.587 |
| Female | 75 (50.0%) | 62 (45.3%) | 73 (51.0%) | 210 (48.8%) |  |
| Male | 75 (50.0%) | 75 (54.7%) | 70 (49.0%) | 220 (51.2%) |  |
| Age [years (SD)] | 63.2 (13.0) | 66.7 (12.4) | 70.9 (12.7) | 66.8 (13.1) | <0.001 |
| Stage [No. %] |  |  |  |  | 0.272 |
| I | 30 (20.0%) | 23 (16.8%) | 20 (14.0%) | 73 (17.0%) |  |
| II | 48 (32.0%) | 57 (41.6%) | 58 (40.6%) | 163 (37.9%) |  |
| III | 50 (33.3%) | 36 (26.3%) | 38 (26.6%) | 124 (28.8%) |  |
| IV | 17 (11.3%) | 17 (12.4%) | 25 (17.5%) | 59 (13.7%) |  |
| Missing | 5 (3.33%) | 4 (2.92%) | 2 (1.40%) | 11 (2.56%) |  |
| MSI Status [Score (SD)] | 0.41 (0.18) | 0.44 (0.25) | 0.44 (0.26) | 0.43 (0.23) | 0.530 |
| Nonsynonymous TMB [Score (SD)] | 14.9 (39.6) | 14.5 (30.6) | 13.7 (25.8) | 14.4 (32.9) | 0.958 |
| 15-gene prognostic group [No. %] |  |  |  |  | 0.163 |
| Good | 53 (35.8%) | 44 (33.1%) | 45 (31.9%) | 142 (33.6%) |  |
| Intermediate | 54 (36.5%) | 46 (34.6%) | 38 (27.0%) | 138 (32.7%) |  |
| Poor | 41 (27.7%) | 43 (32.3%) | 58 (41.1%) | 142 (33.6%) |  |

**Supplementary Table S2: Baseline table of TCGA-STAD cohort.**

|  | **Low** | **Intermediate** | **High** | **Total** | ***P* value** |
| --- | --- | --- | --- | --- | --- |
|  | **N=125** | **N=125** | **N=125** | **N=375** |  |
| Sex [No. %] |  |  |  |  | 0.068 |
| Female | 49 (39.2%) | 33 (26.4%) | 47 (37.6%) | 129 (34.4%) |  |
| Male | 76 (60.8%) | 92 (73.6%) | 78 (62.4%) | 246 (65.6%) |  |
| Age [years (SD)] | 66.3 (11.4) | 66.6 (10.5) | 64.7 (9.82) | 65.9 (10.6) | 0.477 |
| Stage [No. %] |  |  |  |  | 0.001 |
| I | 27 (21.6%) | 15 (12.0%) | 6 (4.80%) | 48 (12.8%) |  |
| II | 34 (27.2%) | 38 (30.4%) | 43 (34.4%) | 115 (30.7%) |  |
| III | 49 (39.2%) | 57 (45.6%) | 63 (50.4%) | 169 (45.1%) |  |
| IV | 14 (11.2%) | 10 (8.00%) | 11 (8.80%) | 35 (9.33%) |  |
| Missing | 1 (0.80%) | 5 (4.00%) | 2 (1.60%) | 8 (2.13%) |  |
| MSI Status [No. %] |  |  |  |  | 0.085 |
| MSS | 66 (52.8%) | 49 (39.2%) | 51 (40.8%) | 166 (44.3%) |  |
| MSI-L | 12 (9.60%) | 6 (4.80%) | 17 (13.6%) | 35 (9.33%) |  |
| MSI-H | 17 (13.6%) | 18 (14.4%) | 10 (8.00%) | 45 (12.0%) |  |
| Missing | 30 (24.0%) | 52 (41.6%) | 47 (37.6%) | 129 (34.4%) |  |
| Nonsynonymous TMB [Score (SD)] | 11.7 (26.4) | 13.5 (24.5) | 7.75 (12.0) | 11.0 (22.2) | 0.260 |
| 53-gene prognostic group [No. %] |  |  |  |  | 0.680 |
| Good | 37 (29.6%) | 39 (31.2%) | 41 (32.8%) | 117 (31.2%) |  |
| Intermediate | 36 (28.8%) | 43 (34.4%) | 44 (35.2%) | 123 (32.8%) |  |
| Poor | 39 (31.2%) | 39 (31.2%) | 30 (24.0%) | 108 (28.8%) |  |
| Missing | 13 (10.4%) | 4 (3.20%) | 10 (8.00%) | 27 (7.20%) |  |

**Supplementary Table S3: Baseline table of TCGA-READ cohort.**

|  | **Low** | **Intermediate** | **High** | **Total** | ***P* value** |
| --- | --- | --- | --- | --- | --- |
|  | **N=53** | **N=54** | **N=50** | **N=157** |  |
| Sex [No. %] |  |  |  |  | 0.495 |
| Female | 23 (43.4%) | 28 (51.9%) | 20 (40.0%) | 71 (45.2%) |  |
| Male | 30 (56.6%) | 26 (48.1%) | 29 (58.0%) | 85 (54.1%) |  |
| Missing | 0 (0.00%) | 0 (0.00%) | 1 (2.00%) | 1 (0.64%) |  |
| Age [years (SD)] | 63.3 (11.4) | 64.1 (12.4) | 64.8 (11.0) | 64.1 (11.5) | 0.835 |
| Stage [No. %] |  |  |  |  | 0.432 |
| I | 13 (24.5%) | 10 (18.5%) | 5 (10.0%) | 28 (17.8%) |  |
| II | 14 (26.4%) | 18 (33.3%) | 16 (32.0%) | 48 (30.6%) |  |
| III | 20 (37.7%) | 14 (25.9%) | 15 (30.0%) | 49 (31.2%) |  |
| IV | 5 (9.43%) | 8 (14.8%) | 9 (18.0%) | 22 (14.0%) |  |
| Missing | 1 (1.89%) | 4 (7.41%) | 5 (10.0%) | 10 (6.37%) |  |
| MSI Status [score (SD)] | 0.34 (0.12) | 0.34 (0.04) | 0.36 (0.15) | 0.35 (0.11) | 0.449 |
| Nonsynonymous TMB [score (SD)] | 15.8 (61.9) | 10.4 (47.8) | 7.67 (24.7) | 11.1 (46.2) | 0.717 |

**Supplementary Table S4: Baseline table of TCGA-ESCA cohort.**

|  | **Low** | **Intermediate** | **High** | **Total** | ***P* value** |
| --- | --- | --- | --- | --- | --- |
|  | **N=67** | **N=37** | **N=52** | **N=156** |  |
| Sex [No. %] |  |  |  |  | 0.246 |
| Female | 8 (11.9%) | 8 (21.6%) | 5 (9.62%) | 21 (13.5%) |  |
| Male | 59 (88.1%) | 29 (78.4%) | 47 (90.4%) | 135 (86.5%) |  |
| Age [years (SD)] | 61.0 (10.6) | 59.2 (12.2) | 61.2 (11.7) | 60.6 (11.3) | 0.672 |
| Stage [No. %] |  |  |  |  | 0.016 |
| I | 8 (11.9%) | 5 (13.5%) | 2 (3.85%) | 15 (9.62%) |  |
| II | 39 (58.2%) | 15 (40.5%) | 13 (25.0%) | 67 (42.9%) |  |
| III | 15 (22.4%) | 12 (32.4%) | 21 (40.4%) | 48 (30.8%) |  |
| IV | 1 (1.49%) | 2 (5.41%) | 4 (7.69%) | 7 (4.49%) |  |
| Missing | 4 (5.97%) | 3 (8.11%) | 12 (23.1%) | 19 (12.2%) |  |
| MSI Status [Score (SD)] | 0.35 (0.13) | 0.33 (0.02) | 0.34 (0.08) | 0.34 (0.10) | 0.735 |
| Nonsynonymous TMB [Score (SD)] | 4.92 (6.77) | 3.81 (2.07) | 4.25 (3.66) | 4.44 (5.03) | 0.538 |
| Lesion site [No. %] |  |  |  |  | 0.243 |
| Proximal | 2 (2.99%) | 3 (8.11%) | 1 (1.92%) | 6 (3.85%) |  |
| Mid | 15 (22.4%) | 13 (35.1%) | 18 (34.6%) | 46 (29.5%) |  |
| Distal | 49 (73.1%) | 21 (56.8%) | 33 (63.5%) | 103 (66.0%) |  |
| Missing | 1 (1.49%) | 0 (0.00%) | 0 (0.00%) | 1 (0.64%) |  |

**Supplementary Table S5: Baseline table of DT-COAD cohort.**

|  | **Low** | **Intermediate** | **High** | **Total** | ***P* value** |
| --- | --- | --- | --- | --- | --- |
|  | **N=29** | **N=100** | **N=114** | **N=243** |  |
| Sex [No. %] |  |  |  |  | 0.786 |
| Female | 13 (44.8%) | 41 (41.0%) | 52 (45.6%) | 106 (43.6%) |  |
| Male | 16 (55.2%) | 59 (59.0%) | 62 (54.4%) | 137 (56.4%) |  |
| Age [years (SD)] | 58.2 (14.7) | 59.4 (12.3) | 59.9 (13.6) | 59.5 (13.2) | 0.830 |
| Stage [No. %] |  |  |  |  | 0.001 |
| I | 0 (0.00%) | 2 (2.00%) | 8 (7.02%) | 10 (4.12%) |  |
| II | 8 (27.6%) | 51 (51.0%) | 73 (64.0%) | 132 (54.3%) |  |
| III | 18 (62.1%) | 41 (41.0%) | 28 (24.6%) | 87 (35.8%) |  |
| IV | 3 (10.3%) | 6 (6.00%) | 5 (4.39%) | 14 (5.76%) |  |
| 15-gene prognostic group [No. %] |  |  |  |  | 0.601 |
| Good  Intermediate Poor | 2 (33.3%) | 14 (27.5%) | 30 (37.0%) | 46 (33.3%) |  |
|  | 2 (33.3%) | 22 (43.1%) | 24 (29.6%) | 48 (34.8%) |  |
|  | 2 (33.3%) | 15 (29.4%) | 27 (33.3%) | 44 (31.9%) |  |

**Supplementary Table S6: Baseline table of DT-READ cohort.**

|  | **Low** | **Intermediate** | **High** | **Total** | ***P* value** |
| --- | --- | --- | --- | --- | --- |
|  | **N=7** | **N=23** | **N=34** | **N=64** |  |
| Sex [No. %] |  |  |  |  | 0.347 |
| Female | 1 (14.3%) | 8 (34.8%) | 15 (44.1%) | 24 (37.5%) |  |
| Male | 6 (85.7%) | 15 (65.2%) | 19 (55.9%) | 40 (62.5%) |  |
| Age [years (SD)] | 54.9 (12.7) | 58.8 (12.5) | 61.4 (11.4) | 59.8 (11.9) | 0.374 |
| Stage [No. %] |  |  |  |  | 0.596 |
| I | 2 (28.6%) | 5 (21.7%) | 5 (14.7%) | 12 (18.8%) |  |
| II | 5 (71.4%) | 18 (78.3%) | 29 (85.3%) | 52 (81.2%) |  |
| 53-gene prognostic group [No. %] |  |  |  |  | 0.987 |
| Good  Intermediate Poor | 2 (28.6%) | 7 (30.4%) | 10 (29.4%) | 19 (29.7%) |  |
|  | 3 (42.9%) | 7 (30.4%) | 12 (35.3%) | 22 (34.4%) |  |
|  | 2 (28.6%) | 9 (39.1%) | 12 (35.3%) | 23 (35.9%) |  |

**Supplementary Table S7: Baseline table of DT-STAD cohort.**

|  | **Low** | **Intermediate** | **High** | **Total** | ***P* value** |
| --- | --- | --- | --- | --- | --- |
|  | **N=162** | **N=36** | **N=10** | **N=208** |  |
| Sex [No. %] |  |  |  |  | 0.277 |
| Female | 41 (25.3%) | 6 (16.7%) | 4 (40.0%) | 51 (24.5%) |  |
| Male | 121 (74.7%) | 30 (83.3%) | 6 (60.0%) | 157 (75.5%) |  |
| Age [years (SD)] | 60.5 (12.3) | 57.8 (11.9) | 67.9 (8.21) | 60.4 (12.2) | 0.067 |
| Stage [No. %] |  |  |  |  | 0.026 |
| I | 13 (8.02%) | 6 (16.7%) | 0 (0.00%) | 19 (9.13%) |  |
| II | 47 (29.0%) | 4 (11.1%) | 0 (0.00%) | 51 (24.5%) |  |
| III | 91 (56.2%) | 25 (69.4%) | 10 (100%) | 126 (60.6%) |  |
| IV | 11 (6.79%) | 1 (2.78%) | 0 (0.00%) | 12 (5.77%) |  |
| 53-gene prognostic group [No. %] |  |  |  |  | 0.946 |
| Good | 49 (30.2%) | 9 (25.0%) | 2 (20.0%) | 60 (28.8%) |  |
| Intermediate | 58 (35.8%) | 14 (38.9%) | 4 (40.0%) | 76 (36.5%) |  |
| Poor | 55 (34.0%) | 13 (36.1%) | 4 (40.0%) | 72 (34.6%) |  |

**Supplementary Table S8: Baseline table of DT-CAP-Discovery cohort.**

|  | **Low** | **Intermediate** | **High** | **Total** | ***P* value** |
| --- | --- | --- | --- | --- | --- |
|  | **N=100** | **N=132** | **N=67** | **N=299** |  |
| Sex [No. %] |  |  |  |  | 0.511 |
| Female | 36 (36.0%) | 41 (31.1%) | 26 (38.8%) | 103 (34.4%) |  |
| Male | 64 (64.0%) | 91 (68.9%) | 41 (61.2%) | 196 (65.6%) |  |
| Age [years (SD)] | 59.0 (11.0) | 59.7 (11.8) | 61.0 (11.5) | 59.8 (11.5) | 0.537 |
| Advanced adenoma [No. %] |  |  |  |  | 0.095 |
| Non-advanced adenoma | 70 (70.0%) | 108 (81.8%) | 49 (73.1%) | 227 (75.9%) |  |
| Advanced adenoma | 30 (30.0%) | 24 (18.2%) | 18 (26.9%) | 72 (24.1%) |  |
| Lesion site [No. %] |  |  |  |  | 0.518 |
| Left Colon | 47 (47.0%) | 50 (37.9%) | 31 (46.3%) | 128 (42.8%) |  |
| Right Colon | 28 (28.0%) | 48 (36.4%) | 18 (26.9%) | 94 (31.4%) |  |
| Whole Colon | 25 (25.0%) | 34 (25.8%) | 18 (26.9%) | 77 (25.8%) |  |
| Total number of adenoma [No. %] |  |  |  |  | 0.030 |
| 1~2 | 87 (87.0%) | 99 (75.0%) | 48 (71.6%) | 234 (78.3%) |  |
| >=3 | 13 (13.0%) | 33 (25.0%) | 19 (28.4%) | 65 (21.7%) |  |

**Supplementary Table S9: Baseline table of DT-CAP-Validation cohort.**

|  | **Low** | **Intermediate** | **High** | **Total** | ***P* value** |
| --- | --- | --- | --- | --- | --- |
|  | **N=122** | **N=309** | **N=150** | **N=581** |  |
| Sex [No. %] |  |  |  |  | 0.011 |
| Female | 52 (42.6%) | 120 (38.8%) | 40 (26.7%) | 212 (36.5%) |  |
| Male | 70 (57.4%) | 189 (61.2%) | 110 (73.3%) | 369 (63.5%) |  |
| Age [years (SD)] | 57.0 (12.2) | 58.3 (12.1) | 61.7 (10.7) | 58.9 (11.9) | 0.002 |
| Advanced adenoma [No. %] |  |  |  |  | <0.001 |
| Non-advanced adenoma | 81 (66.4%) | 256 (82.8%) | 104 (69.3%) | 441 (75.9%) |  |
| Advanced adenoma | 41 (33.6%) | 53 (17.2%) | 46 (30.7%) | 140 (24.1%) |  |
| Lesion site [No. %] |  |  |  |  | <0.001 |
| Left Colon | 59 (48.4%) | 131 (42.4%) | 47 (31.3%) | 237 (40.8%) |  |
| Right Colon | 43 (35.2%) | 130 (42.1%) | 53 (35.3%) | 226 (38.9%) |  |
| Whole Colon | 20 (16.4%) | 48 (15.5%) | 50 (33.3%) | 118 (20.3%) |  |
| Total number of adenoma [No. %] |  |  |  |  | 0.004 |
| 1~2 | 97 (79.5%) | 258 (83.5%) | 105 (70.0%) | 460 (79.2%) |  |
| >=3 | 25 (20.5%) | 51 (16.5%) | 45 (30.0%) | 121 (20.8%) |  |

**Supplementary Table S10: Baseline table of combined DT-CAP cohort.**

|  | **Discovery** | **Validation** | **Total** | ***P* value** |
| --- | --- | --- | --- | --- |
|  | **N=299** | **N=581** | **N=880** |  |
| Sex [No. %] |  |  |  | 0.600 |
| Female | 103 (34.4%) | 212 (36.5%) | 315 (35.8%) |  |
| Male | 196 (65.6%) | 369 (63.5%) | 565 (64.2%) |  |
| Age [years (SD)] | 59.8 (11.5) | 58.9 (11.9) | 59.2 (11.7) | 0.287 |
| Follow-up duration [years (SD)] | 26.2 (12.6) | 26.7 (12.4) | 26.6 (12.4) | 0.557 |
| Advanced adenoma [No. %] |  |  |  | 1.000 |
| Non-advanced adenoma | 227 (75.9%) | 441 (75.9%) | 668 (75.9%) |  |
| Advanced adenoma | 72 (24.1%) | 140 (24.1%) | 212 (24.1%) |  |
| Lesion site [No. %] |  |  |  | 0.054 |
| Left Colon | 128 (42.8%) | 237 (40.8%) | 365 (41.5%) |  |
| Right Colon | 94 (31.4%) | 226 (38.9%) | 320 (36.4%) |  |
| Whole Colon | 77 (25.8%) | 118 (20.3%) | 195 (22.2%) |  |
| Total number of adenoma [No. %] |  |  |  | 0.820 |
| 1~2 | 234 (78.3%) | 460 (79.2%) | 694 (78.9%) |  |
| >=3 | 65 (21.7%) | 121 (20.8%) | 186 (21.1%) |  |

**Supplementary Table S11: Baseline table of DT-EEL cohort.**

|  | **Low** | **Intermediate** | **High** | **Total** | ***P* value** |
| --- | --- | --- | --- | --- | --- |
|  | **N=46** | **N=45** | **N=45** | **N=136** |  |
| Sex [No. %] |  |  |  |  | 0.156 |
| Female | 19 (41.3%) | 13 (28.9%) | 12 (26.7%) | 44 (32.4%) |  |
| Male | 23 (50.0%) | 30 (66.7%) | 33 (73.3%) | 86 (63.2%) |  |
| Missing | 4 (8.70%) | 2 (4.44%) | 0 (0.00%) | 6 (4.41%) |  |
| Age [years (SD)] | 59.7 (11.0) | 62.2 (9.08) | 64.6 (8.85) | 62.2 (9.82) | 0.056 |
| Lesion site [No. %] |  |  |  |  | 0.136 |
| Upper esophagus | 4 (8.70%) | 1 (2.22%) | 6 (13.3%) | 11 (8.09%) |  |
| Middle esophagus | 25 (54.3%) | 32 (71.1%) | 30 (66.7%) | 87 (64.0%) |  |
| Lower esophagus | 17 (37.0%) | 12 (26.7%) | 9 (20.0%) | 38 (27.9%) |  |
| Initial diagnosis [No. %] |  |  |  |  | 0.004 |
| Normal | 0 (0.00%) | 2 (4.44%) | 1 (2.22%) | 3 (2.21%) |  |
| Inflammation | 42 (91.3%) | 26 (57.8%) | 28 (62.2%) | 96 (70.6%) |  |
| LGIN | 3 (6.52%) | 14 (31.1%) | 11 (24.4%) | 28 (20.6%) |  |
| HGIN | 1 (2.17%) | 3 (6.67%) | 5 (11.1%) | 9 (6.62%) |  |

**Supplementary Table S12: Baseline table of DT-COAD scRNAseq cohort.**

|  | **Low** | **Intermediate** | **High** | **Total** | ***P* value** |
| --- | --- | --- | --- | --- | --- |
|  | **N=10** | **N=4** | **N=6** | **N=20** |  |
| Sex [No. %] |  |  |  |  | 0.632 |
| Male | 4 (40.0%) | 3 (75.0%) | 3 (50.0%) | 10 (50.0%) |  |
| Female | 6 (60.0%) | 1 (25.0%) | 3 (50.0%) | 10 (50.0%) |  |
| Age [years (SD)] | 61.2 (15.8) | 74.0 (8.00) | 63.2 (10.7) | 64.3 (13.6) | 0.284 |
| Stage [No. %] |  |  |  |  | 0.733 |
| I | 1 (10.0%) | 0 (0.00%) | 0 (0.00%) | 1 (5.00%) |  |
| II | 4 (40.0%) | 2 (50.0%) | 1 (16.7%) | 7 (35.0%) |  |
| III | 4 (40.0%) | 1 (25.0%) | 2 (33.3%) | 7 (35.0%) |  |
| IV | 1 (10.0%) | 1 (25.0%) | 3 (50.0%) | 5 (25.0%) |  |
| MSI_status [No.] |  |  |  |  | 0.500 |
| MSI | 0 (0.00%) | 0 (0.00%) | 1 (16.7%) | 1 (5.00%) |  |
| MSS | 10 (100%) | 4 (100%) | 5 (83.3%) | 19 (95.0%) |  |

**Supplementary Table S13.** CMB Coefficients for Cancer-Specific CMBRS Calculation.

| **CMB** | **COAD** | **READ** | **STAD** | **ESCA** |
| --- | --- | --- | --- | --- |
| CMB_76.category | -2.36206 | -0.20417 | -0.05372 | 0.341908 |
| CMB_97.category | 0.796055 | -1.65934 | 0.391993 | 0.763568 |
| CMB_9.category | 1.239994 | 0.464021 | -0.18901 | -0.29099 |
| CMB_29.category | 1.167279 | 1.06299 | -0.24949 | -0.26966 |
| CMB_12.category | -1.40122 | 15.66748 | -0.29193 | 0.992964 |
| CMB_87.category | 0.7735 | -0.7763 | 0.232599 | 0.415056 |
| CMB_67.category | 1.675393 | -17.0766 | -0.20035 | -0.34131 |
| CMB_120.category | -1.05369 | 0.419921 | 0.206797 | 0.94114 |
| CMB_86.category | 0.971109 | 0.358993 | 0.527741 | -0.15628 |
| CMB_30.category | -1.09462 | -0.18762 | 0.544172 | -0.04367 |
| CMB_85.category | -0.98127 | 1.144929 | -0.13794 | -0.06023 |
| CMB_47.category | 1.620678 | -0.31245 | 0.452912 | -0.86275 |
| CMB_104.category | 0.850017 | 0.864199 | 0.085013 | 0.65273 |

**Supplementary Table S14. Comparison of performance between multimodal integration of markers and clinical markers.**

| **Performance increase (%)**  **compared to baseline model (e.g., model built on clinical factors)** | **Cancers** | | | | | | **Pre-cancerous lesions** | | |
| --- | --- | --- | --- | --- | --- | --- | --- | --- | --- |
|  | **Public cohorts** | | | | **Independent hospital cohorts** | | | | |
|  | TCGA-COAD | TCGA-READ | TCGA-STAD | TCGA-ESCA | DT-COAD/READ | DT-STAD | DT-CAP-discovery | DT-CAP-validation | DT-EEL |
| CMB + Clinical factors | 16.2% | 33.7% | 10.4% | 9.2% | 17.0% | 6.6% | 14.3% | 5.0% | 49.6% |
| CMB + Gene + Clinical factors | 18.3% | NA | 19.6% | NA | 33.6% | 8.1% | NA | NA | NA |

**Supplementary Table S15.** List of CMB-associated genes. (see separate excel file)

**Supplementary Data S1-S10.** Clinical data, along with CMBs, CMBs’ categorical classifications, CMBRS, and CMBRG in the TCGA and Drum Tower cohorts. (see separate excel file)
